# Monitoring Resistance and Biochemical Studies of Three Egyptian Field Strains of *Spodoptera littoralis* (Lepidoptera: Noctuidae) to Six Insecticides

**DOI:** 10.3390/toxics11030211

**Published:** 2023-02-24

**Authors:** Moataz A. M. Moustafa, Rasha I. A. Moteleb, Yehia F. Ghoneim, Sameh Sh. Hafez, Reham E. Ali, Essam E. A. Eweis, Nancy N. Hassan

**Affiliations:** 1 Department of Economic Entomology and Pesticides, Faculty of Agriculture, Cairo University, Giza 12613, Egypt; 2 Department of Insecticide Resistance, Central Agricultural Pesticides Laboratory, Agricultural Research Center, Giza 12618, Egypt

**Keywords:** resistance, susceptibility, monitoring, *Spodoptera littoralis*, insecticides, detoxification enzymes

## Abstract

Background: *Spodoptera littoralis* (Boisd.) is a prominent agricultural insect pest that has developed resistance to a variety of insecticide classes. In this study, the resistance of three field strains of *S. littoralis,* collected over three consecutive seasons (2018 to 2020) from three Egyptian Governorates (El-Fayoum, Behera and Kafr El-Shiekh), to six insecticides was monitored. Methods: Laboratory bioassays were carried out using the leaf-dipping method to examine the susceptibility of the laboratory and field strains to the tested insecticides. Activities of detoxification enzymes were determined in an attempt to identify resistance mechanisms. Results: The results showed that LC_50_ values of the field strains ranged from 0.0089 to 132.24 mg/L, and the corresponding resistance ratio (RR) ranged from 0.17 to 4.13-fold compared with the susceptible strain. Notably, low resistance developed to spinosad in all field strains, and very low resistance developed to alpha-cypermethrin and chlorpyrifos. On the other hand, no resistance developed to methomyl, hexaflumeron or *Bacillus thuringiensis*. The determination of detoxification enzymes, including carboxylesterases (α- and β-esterase), mixed function oxidase (MFO) and glutathione-*S*-transferase (GST), or the target site of acetylcholinesterase (AChE), revealed that the three field strains had significantly different activity levels compared with the susceptible strain. Conclusion: Our findings, along with other tactics, are expected to help with the resistance management of *S. littoralis* in Egypt.

## 1. Introduction

The cotton leaf worm *Spodoptera littoralis* (Boisd.) is an insect, which causes serious losses in over 80 economically significant crop species [1]. It is spread in Africa and in the Middle Eastern nations [2]. Excessive dependence on chemical control has led to the development of resistance to numerous classes of insecticides, and insect resistance to insecticides has been documented in more than 600 arthropod species [3].

Resistance was first reported in *S. littoralis* in 1968 to methyl-parathion, which belongs to organophosphates [1]. In recent years, *S. littoralis* has evolved high levels of resistance to different groups of insecticides, such as organophosphates, pyrethroids, carbamates and insect growth regulators (IGRs) [4,5], as well as to several newer insecticides, including indoxacarb and chlorantraniliprole [6,7]. In addition, *S. littoralis* is resistant to bioinsecticides, including *Bacillus thuringiensis* [5], spinosad [7,8] and spinotram [7], due to the acceleration caused by the absence of a hibernation period in this pest [9]. Currently, *S. littoralis* is in the top 30 most resistant species in the world, according to the Arthropod Pesticide Resistance Database (http://www.pesticideresistance.org, accessed on 18 May 2021). Thus, other species, such as *Spodoptera litura* (Fabricius) [10,11,12], *Spodoptera frugiperda* (Smith) [13] and *Spodoptera exigua* (Hübner) [14], have developed resistance to a variety of insecticide groups due to long-term insecticide application.

Two major mechanisms of insecticide resistance are known [3], including target change and mechanisms that reduce the amount of insecticide reaching the target (reduced penetration and interference of detoxification enzymes). Target site resistance mediated by acetylcholinesterase enzyme (AChE) insensitivity to insecticides has been investigated by biochemical approaches in several Spodoptera species [15,16,17]. In addition, metabolic detoxification enzymes including carboxylesterases (CarEs), mixed function oxidase (MFO) and glutathione S-transferases (GSTs) are remarkable defensive physiological reactions, which are essential for insecticide metabolism [18,19].

Both CarEs and MFO are mediated reactions [20], resulting in the reduction in or oxidation of chemical insecticides. GSTs convert the detoxified molecules into more water-soluble forms by glutathione conjugation, which facilitates their rapid removal from the cell [21]. This could be achieved by over-expression [22] or expressing duplicated isoforms of these enzymes [23].

Consequently, understanding resistance to insecticides and its mechanisms could improve resistance management strategies. This understanding can be achieved by monitoring the metabolic enzymes’ activity as an efficient biomarker [24,25], whether in the region or between regions. Generally, effective resistance monitoring depends on the availability of reliable methods of monitoring, including the bioassay and biochemical methods that represent the primary means of testing for insecticide resistance [1].

Therefore, in the present study, the status of resistance to six insecticides (chlorpyrifos, methomyl, alpha-cypermethrin, hexaflumeron, *Bacillus thuringiensis* and spinosad) has been monitored in three field strains collected from three Egyptian governorates (El-Fayoum, Beheira and Kafr El-Shiekh) over three consecutive seasons (2018 to 2020). In addition, the activity of detoxification enzymes was investigated to explore the mechanism of resistance to the tested compounds.

## 2. Materials and Methods

### 2.1. Insects

A susceptible laboratory strain of *S. littoralis* was continuously inbred on castor bean (Ricinus communis) leaves under laboratory conditions [7,26] for more than ten generations with no exposure to any pesticides. The adult moths fed on sugar solution (10%) as a dietary supplement [27]. Three field strains of *S. littoralis* were collected from three Egyptian governorates (Fayoum; 29°18′30″ N and 30°50′39″ E, Beheira; 30°59′00″ N and 30°12′00″ E and Kafr-El-Shiekh; 31°06′42″ N and 30°56′45″ E) over three consecutive seasons (2018 to 2020). Egg masses of *S. littoralis* were collected from cotton and vegetable fields in May–July, before pesticide application. These masses were maintained in a rearing room at 60–70% relative humidity, at a temperature of 25 ± 1 °C and a 16 h:8 h light/dark regimen, to obtain the 4th instar larvae for bioassays and biochemical studies.

### 2.2. Insecticides and Reagents Used

The insecticides used in bioassays are presented in Table 1. The substrates and reagents used for biochemical studies were purchased from Sigma Aldrich, Germany.

### 2.3. Bioassay

The leaf-dipping method was conducted as described by Moustafa et al. [29] and Awad et al. [30]. Fourth-instar larvae of *S. littoralis* were used for the bioassays. As shown in Table S1, 6 concentrations, ranging from 0.002 to 16 mg/L, of each tested insecticide were used. Castor bean leaves were immersed in each concentration for 20 s; untreated leaves were immersed in water for the control group then t allowed to air-dry. Leaves with ten larvae were then transferred into a glass container (0.25 L), and five replicates were performed for each concentration [7,31]. For conventional insecticides, i.e., chlorpyrifos, methomyl and alpha-cypermethrin, lethal concentrations (LCs) were calculated after 24 h of treatment. Meanwhile, for bioinsecticides, i.e., hexaflumeron, *B. thuringiensis* and spinosad, the larvae fed on insecticide-treated leaves for one day, then on untreated leaves for two days, and mortality was recorded by the end of the third day to calculate LC_50_ and LC_90_.

### 2.4. Biochemical Analysis

#### 2.4.1. Sample Preparation

For enzyme activity assays, 50 mg of untreated fourth instar larvae from susceptible or field strains were homogenized in 1 mL distilled water in a chilled glass Teflon tissue homogenizer. Homogenates were centrifuged at 8000 rpm for 15 min at 4 °C. The supernatants were kept at −20 °C prior to biochemical assays. Three replicates were used for each strain. Protein content was determined as described by Bradford [32].

#### 2.4.2. Detoxification Enzyme Assays

##### Carboxylesterase (CarE)

Determination of α- and β-esterases’ activity was performed as described by Van Asperen [33]. The hydrolysis of α- or β-naphthyl acetate was spectrophotometrically measured at 600 and 550 nm, respectively. The total CarE activity was calculated using the standard curves of α- and β-naphthol and protein content.

##### Mixed Function Oxidase (MFO)

The activity of MFO was measured as described by Hansen and Hodgson [34]. The reaction mixture of enzyme solution, NADPH, glucose-6-phosphate and Glucose-6-phosphate dehydrogenase was initiated by adding p-nitroanisole and incubated at 37 °C for 30 min. The reaction was then terminated by adding HCl. The optical density was measured spectrophotometrically for 10 min at 405 nm.

##### Glutathione S-Transferase (GST)

The activity of GST was determined according to Habig et al. [35]. 1-chloro 2,4-dinitrobenzene (CDNB) was used as a substrate. The mixture of potassium phosphate buffer, GSH, enzyme solution and the substrate was incubated for 5 min at 30 °C. The absorbance increase at 340 nm was then recorded versus a blank mixture. The nanomole-conjugated substrate/min/mg protein was then determined.

#### 2.4.3. Acetylcholine Esterase (AChE)

The activity of AChE was determined as indicated by Simpson et al. [36]. Acetylcholine bromide (AChBr) was used as a substrate. The reaction mixture of the enzyme solution, phosphate buffer and substrate was incubated for 30 min at 37 °C. The AChBr decrease was read at 515 nm.

### 2.5. Statistical Analysis

Probit analysis was used to calculate median lethal concentrations (LC_50_) and their 95% confidence limits (CLs) [37]. To calculate the resistance ratio (RR), the LC_50_ of a field strain was divided by the LC_50_ of the laboratory strain. In addition, one-way ANOVA with GraphPad-Prism v. 9.3 statistical analysis software were used to analyze the enzymes’ activity data. Differences among the means of strains were assessed using Tukey’s HSD test at a significance level of *p* ≤ 0.05.

## 3. Results

### 3.1. Susceptibility of Laboratory Strain of S. littoralis to the Tested Insecticides

The toxicity levels of the tested insecticides (chlorpyrifos, methomyl, alpha-cypermethrin, hexaflumeron, *B. thuringiensis* and spinosad) to the susceptible strain of *S. littoralis* are shown in Table 2. Overall, based on LC_50_ values, insecticide toxicity to *S. littoralis* was as follows, in descending order: Spinosad > *B. thuringiensis* > hexaflumeron > alpha-cypermethrin > chlorpyrifos > methomyl. It is obvious that the bioinsecticides (spinosad, *B. thuringiensis* and hexaflumeron) were more toxic than the conventional ones, with LC_50_ of 0.0089, 6.11 and 14.57 mg/L, respectively.

### 3.2. Susceptibility of Field Strains of S. littoralis to Conventional Insecticides

As shown in Table 3, Table 4 and Table 5, in 2018, the Fayoum and Kafr El-Shiekh strains of *S. littoralis* showed very low resistance levels to chlorpyrifos (2.37- and 2.08-fold, respectively). In 2019, the resistance ratios were 1.19, 1.38 and 1.18-fold for the Fayom, Beheira and Kafr El-Shiekh strains, respectively. In 2020, resistance to chlorpyrifos decreased to 1.10- and 0.65-fold in Beheira and Kafr El-Shiekh strains, respectively). As to methomyl, very low levels of resistance to it were observed in the Fayoum strain (3.11-fold) in 2020 (Table 3) and in Beheira strain (2.31-fold) in 2018 (Table 4). In contrast, a resistance ratio of <2-fold was found in the Kafr El-Sheikh strain (Table 5). Regarding alpha-cypermethrin, a very low level of resistance to it was found in the Fayoum strain (2.02-fold) in 2018 (Table 3); in the Beheira strain (2.65- and 2.08-fold) in 2018 and 2019, respectively (Table 4); and in the Kafr El-Sheikh strain (2.79-fold) in 2019 (Table 5).

### 3.3. Susceptibility of Field Strains of S. littoralis to Bioinsecticides

The data showed no resistance to hexaflumeron in any of the field strains (Table 3, Table 4 and Table 5) during the three seasons (2018–2020), and the resistance ratios ranged from 0.07- to 0.31-fold. Regarding *B. thuringiensis,* no resistance was detected in the Fayoum and Bereira strains (Table 3 and Table 4), while a moderate level of resistance (2.19-fold) was recorded in the Kafr El-Shiekh strain in 2020 (Table 5). Nevertheless, a low level of resistance to spinosad was observed in all field strains (Table 3, Table 4 and Table 5), and the resistance ratios ranged from 1.79- to 4.31-fold.

### 3.4. Activity of Detoxification Enzymes

To examine the prospective role of detoxification enzymes in the susceptibility of *S. littoralis* to the tested insecticides, enzymes assays were performed to determine the levels of carboxylesterases (α and β- esterases), acetylcholine esterase (AChE), glutathione-S-transferase (GST) and mixed-function oxidase (MFO) in the tested field strains in comparison with the laboratory one. Data are shown in Table 6 and Table 7.

As shown in Table 6, the level of α-esterase, expressed as folds of that of the susceptible strain, was reduced to 0.96-, 0.92- and 0.79-fold in Fayoum strain; to 0.89-, 0.85- and 0.88-fold in Beheira strain; and to 0.81-, 0.92- and 0.93-fold in the Kafr El-Sheikh strain over the three seasons, respectively. A similar reduction was also recorded for the level of β-esterases enzyme. In addition, AchE significantly decreased in the Beheira strain in all seasons, but only in 2020 for the Fayoum strain, and in 2018 for the Kafr El-Sheikh strain.

As shown in Table 7, the levels of MFO and GST significantly decreased in the Beheira strain in all seasons. MFO levels ranged from 1.26 to 1.32 mg/mg of protein, while GST ranged from 1.92 to 2.01 mmol/min/mg of protein. Similarly, the level of both enzymes significantly decreased, but only in 2020 for the Fayoum strain and in 2018 for the Kafr El-Sheikh strain.

## 4. Discussion

The indiscriminate use of conventional and newer insecticides has caused the development of resistance to almost all kinds of insecticides in the Noctuidae species [7,12,13,38,39,40,41]. According to the Egyptian Agricultural Pesticides Committee, EAPC (2022), several groups of biochemical or chemical insecticides, such as organophosphorus, pyrethroids, insect growth regulators (IGRs), diamides, oxadiazin, spinosyns, emamectin benzoate and *B. thuringiensis*, are used for *S. littoralis* management. Hence, insecticide resistance has developed in this insect pest [7,42,43]. Consequently, the history of insect resistance to various insecticides, including *S. littoralis,* should be studied to monitor the tolerance changes and detect any problems that may occur. In addition, continuous monitoring of the insect strains for changes in resistance frequencies is needed for the development of effective management strategies [44]. Therefore, monitoring insecticides is considered a pre-requisite in IPM programs [45], and becomes a remarkable aspect of resistance management [3]. The present study investigated the susceptibility of the fourth instar larvae of three field strains of *S. littoralis* to six insecticides with different modes of action over three consecutive seasons (2018–2020). To explore the mechanism of resistance, if it existed, the activities of the relevant enzymes were also studied.

The results showed that bioinsecticides were more toxic to *S. littoralis* than conventional ones. Spinosad had the highest toxicity, with an LC_50_ value of 0.0089 mg/L. These results are congruent with previous studies by Ahmed et al. [8] and Tamilselvan et al. [46], who found that spinosad and other bioinsecticides, such as emamectin benzoate and spinotram, were more toxic to *S. littoralis* and *Plutella xylostella* (L.) than conventional insecticides. Based on the resistance ratio, all field strains developed resistance to spinosad compared with other insecticides. In line with this finding, resistance to spinosad has been reported in several insect pests, including *S. littoralis* [7,8], *S. litura* [19], *S. exigua* [14] and *P. xylostella* [46]. On the contrary, a slight level of resistance was developed to chlorpyrifos (organophosphorus) and methomyl (carbamates) in some cases, but in others, no resistance was found. These findings can be attributed to the fact that in Egypt, bioinsecticides, including spinosad and newer chemical insecticides, have been extensively used due to the health and environmental issues associated with conventional ones. The fluctuation of resistance levels recorded in this study can be connected with the type of insecticides used and the sequence of usage in each governorate.

Metabolic resistance relies on enzymatic systems that can detoxify and/or sequester toxic molecules, interrupting or decreasing its harmful effect [47]. Mostly, metabolic resistance, which involves three major families of enzymes (CarE, MFO and GST), is one of the most common defense mechanisms in insects [7,25,48,49]. The point mutation at the target site is involved in the insect resistance mechanism, like that of AChE, which is involved in organophosphates and carbamates insecticides’ resistance [50]. Therefore, the activity of these enzymes together with AChE was determined in the three field strains of *S. littoralis* to assess their roles in resistance and to identify resistance mechanisms for the sake of enlightened pest management. In this regard, our results revealed, unexpectedly, a reduction in the activities of all enzymes in field strains. This might suggest that the resistance of these strains to the tested insecticides is not always associated with higher detoxification activity, but is, rather, related to a different mechanism. These results are not consistent with those of Hu et al. [51] and Zhang et al. [52], who found that the overexpression of CarE and GST was related to resistance to insecticides. In fact, most of the resistance to insecticides is associated with an increase in the activity of detoxification enzymes [7,25,53,54]. However, modification of the target site could lead to insensitivity of insect pests to insecticides [55]. In addition, factors such as UV light, sunlight, photolysis in water and shelf life could affect the insecticide efficiency and delay the development of the resistance/prevent it from occurring [56]. Thus, we speculate that the inconsistency with other studies might be due to the different species of insect, type of insecticide, time of sampling or method of treatment.

Moreover, studies have confirmed cross-resistance between spinosad and newer chemical insecticides [46,52,57]. Cross-resistance between different groups of insecticides might be due to metabolic detoxification mechanisms [58]. Therefore, it is necessary to develop effective management plans to delay any resistance development.

## 5. Conclusions

In summary, our study provides evidence of very low to non-resistance development by *S. littoralis* to some commonly used insecticides in some Egyptian governorates due to the successful resistance management strategies used in Egypt. However, monitoring resistance to insecticides is an important aspect of insecticide rotation and their mixed application. Thus, regular follow up is needed to specify and confirm the mechanisms by which *S. littoralis* develops resistance to the tested insecticides, in order to avoid resistance problems and pest control failure.

## Figures and Tables

**Table 1:** Used insecticides and their actions.

Common Name	Trade Name	(a.i. %) Formulation	Mode of Action *
Chlorpyrifos	Dursban	48% EC	Acetylcholinesterase (AChE) inhibitors
Methomyl	Methomyl	90% WP	
Alpha-cypermethrin	Alpha-cypermethrin	10% EC	Sodium channel modulators
Hexaflumeron	Demeron	10% EC	Inhibitors of chitin biosynthesis
*Bacillus thuringiensis*	Dipel-2X	6.4% WP	Microbial disruptors of insect midgut membranes
Spinosad	Spinotor	24% SC	Nicotinic acetylcholine receptor (nAChR) allosteric modulators—Site I

* Insecticides Resistance Action Committee, IRAC [28].

**Table 2:** Susceptibility of the 4th instar larvae of a laboratory strain of *S. littoralis* to the tested insecticides. Castor leaves were dipped in six concentrations of each tested insecticide. Five replicates, ten larvae each, were used for each concentration. (i.e., 50 larvae/concentration). The larvae were allowed to feed for 24 h on leaves treated with conventional insecticides, and for 72 h post-treatment with bioinsecticides. LC_50_ and LC_90_ values of the tested insecticides were calculated using the (LdPLine©) package software according to the Log-Probit analysis method.

Insecticide	^a^ LC_50_ (mg/L) (95% Confidence Limits)	^b^ LC_90_ (mg/L) (95% Confidence Limits)	Slope ± SE	χ^2^
Chlorpyrifos	86.04 (54.44–124.08)	760.34 (408.06–2807.41)	1.35 ± 0.26	0.12
Methomyl	132.24 (53.41–208.59)	1243.29 (739.91–3809.46)	1.31 ± 0.29	1.10
Alpha-cypermethrin	48.78 (25.53–72.79)	502.78 (270.51–1989.44)	1.26 ± 0.27	1.92
Hexaflumeron	14.57 (7.51–22.38)	196.49 (91.78–1275.15)	1.13 ± 0.26	0.71
*B. thuringiensis*	6.11 (0.91–11.90)	113.62 (57.63–802.94)	1.00 ± 0.28	0.49
Spinosad	0.0089 (0.007–0.011)	0.035 (0.02–0.08)	2.14 ± 0.39	2.61

^a^ LC_50_: concentration causing 50% mortality. ^b^ LC_90_: concentration causing 90% mortality.

**Table 3:** Susceptibility of fourth instar larvae of Fayoum field strain of *Spodoptera littoralis* to six insecticides over three consecutive seasons (2018 to 2020). Egg masses of *S. littoralis*, collected over three consecutive seasons (2018 to 2020), were kept in the rearing room until the fourth instar larvae. Six concentrations of each tested insecticide were prepared, then castor bean leaves were immersed in each concentration for 20 s. For each concentration, 50 larvae were used to calculate the LC_50_, LC_90_ and the resistance ratio.

Insecticides	Season	^a^ LC_50_ (mg/L) (95% Confidence Limit)	^b^ LC_90_ (mg/L) (95% Confidence Limit)	Slope ± SE	χ^2^	^c^ RR
Chlorpyrifos	2018	204.31 (150.29–275.72)	965.09 (610.59–2245.77)	1.90 ± 0.32	6.40	2.37
	2019	102.69 (74.77–137.92)	543.90 (344.54–1223.02)	1.77 ± 0.28	4.04	1.19
	2020	168.30 (120.04–224.41)	869.28 (566.75–1841.26)	1.79 ± 0.29	2.70	1.95
Methomyl	2018	193.17 (122.05–264.07)	1019.42 (686.81–2057.66)	1.77 ± 0.31	0.20	1.46
	2019	147.13 (104.34–195.59)	741.26 (490.02–1524.05)	1.82 ± 0.29	0.88	1.11
	2020	411.12 (276.36–753.85)	4371.65 (1763.31–36986.34)	1.24 ± 0.26	0.58	3.11
Alpha-cypermethrin	2018	98.74 (79.94–121.31)	293.46 (223.13–438.38)	2.70 ± 0.33	0.64	2.02
	2019	87.95 (65.07–116.97)	432.67 (280.17–920.30)	1.85 ± 0.29	5.68	1.80
	2020	24.34 (13.37–35.65)	220.31 (124.36–738.47)	1.33 ± 0.27	0.19	0.50
Hexaflumeron	2018	3.19 (0.65–7.93)	742.90 (154.31–43013.25)	0.54 ± 0.13	0.79	0.22
	2019	1.06 (0.11–2.89)	156.31 (44.11–3986.73)	0.59 ± 0.14	1.85	0.07
	2020	2.08 (0.61–4.38)	117.30 (43.44–872.58)	0.73 ± 0.14	0.62	0.14
*B. thuringiensis*	2018	6.58 (2.37–13.27)	377.19 (133.40–2969.85)	0.72 ± 0.14	0.71	1.08
	2019	6.009 (2.22–11.88)	289.44 (109.52–1885.72)	0.76 ± 0.14	0.23	0.98
	2020	4.79 (1.65–9.61)	221.02 (85.80 -1385.08)	0.77 ± 0.14	0.32	0.78
Spinosad	2018	0.015 (0.0051–0.032)	0.75 (0.30–4.40)	0.76 ± 0.14	0.57	1.69
	2019	0.036 (0.015–0.071)	1.80 (0.64–13.23)	0.75 ± 0.13	0.20	4.04
	2020	0.026 (0.009–0.054)	1.77 (0.59–16.45)	0.70 ± 0.13	1.22	2.92

^a^ LC_50_: concentration causing 50% mortality. ^b^ LC_90_: concentration causing 90% mortality. ^c^ RR: LC_50_ value of the field strain/LC_50_ value of the susceptible strain.

**Table 4:** Susceptibility of the fourth instar larvae of Beheira field strain of *Spodoptera littoralis* to six insecticides over three consecutive seasons (2018 to 2020). Egg masses of *S. littoralis*, collected over three consecutive seasons (2018 to 2020), were kept in the rearing room until the fourth instar larvae. Six concentrations of each tested insecticide were prepared, then castor bean leaves were immersed in each concentration for 20 s. For each concentration, 50 larvae were used to calculate the LC_50_, LC_90_ and the resistance ratio.

Insecticide	Season	^a^ LC_50_ (mg/L) (95% Confidence Limit)	^b^ LC_90_ (mg/L) (95% Confidence Limit)	Slope ± SE	χ^2^	^c^ RR
Chlorpyrifos	2018	72.55 (48.41–99.68)	431.41 (266.53–1085.73)	1.65 ± 0.30	1.93	0.84
	2019	119.06 (93.80–151.03)	409.99 (293.39–696.98)	2.38 ± 0.33	1.04	1.38
	2020	94.78 (44.95–144.06)	948.82 (532.63–3380.33)	1.281 ± 0.27	0.21	1.10
Methomyl	2018	350.19 (242.53–573.08)	3134.30 (1435.68–17256.54)	1.34 ± 0.26	1.27	2.65
	2019	275.24 (209.89–369.16)	1246.54 (799.69–2682.82)	1.95 ± 0.29	4.56	2.08
	2020	75.85 (26.33–124.87)	1102.79 (554.11–6393.61)	1.10 ± 0.27	0.31	0.57
Alpha-cypermethrin	2018	112.54 (83.95–153.34)	582.37 (359.57–1377.15)	1.79 ± 0.28	1.96	2.31
	2019	47.36 (33.46–66.34)	315.67 (181.29–914.35)	1.55 ± 0.27	1.35	0.97
	2020	20.78 (10.50–30.97)	183.69 (103.24–669.79)	1.35 ± 0.29	2.53	0.43
Hexaflumeron	2018	4.47 (1.13–10.88)	1000.84 (197.07–64021.65)	0.54 ± 0.13	0.35	0.31
	2019	2.27 (0.35–5.90)	579.16 (123.98–33838.96)	0.53 ± 0.13	0.41	0.16
	2020	4.45 (1.55–9.40)	381.00 (112.35–5137.51)	0.66 ± 0.13	0.84	0.31
*B. thuringiensis*	2018	5.02 (1.54–10.61)	317.07 (105.57–3351.50)	0.71 ± 0.14	1.92	0.82
	2019	9.38 (3.45–19.56)	748.66 (223.46–9533.92)	0.67 ± 0.13	0.52	1.54
	2020	5.83 (2.03–11.81)	325.57 (117.90–2424.64)	0.73 ± 0.14	0.11	0.95
Spinosad	2018	0.034 (0.011–0.076)	4.14 (1.06–90.99)	0.61 ± 0.13	1.06	3.82
	2019	0.022 (0.007–0.045)	1.34 (0.47–10.77)	0.72 ± 0.14	0.75	2.47
	2020	0.030 (0.008–0.070)	4.83 (1.12–156.15)	0.58 ± 0.13	0.88	3.37

^a^ LC_50_: concentration causing 50% mortality. ^b^ LC_90_: concentration causing 90% mortality. ^c^ RR: LC_50_ value of the field strain/LC_50_ value of the susceptible strain.

**Table 5:** Susceptibility of the fourth instar larvae of Kafr El-Shiekh field strain of *Spodoptera littoralis* to six insecticides over three consecutive seasons (2018 to 2020). Egg masses of *S. littoralis*, collected over three consecutive seasons (2018 to 2020), were kept in the rearing room until the fourth instar larvae. Six concentrations of each tested insecticide were prepared, then castor bean leaves were immersed in each concentration for 20 s. For each concentration, 50 larvae were used to calculate the LC_50_, LC_90_ and the resistance ratio.

Insecticide	Season	^a^ LC_50_ (mg/L) (95% Confidence limit)	^b^ LC_90_ (mg/L) (95% Confidence limit)	Slope ± SE	χ^2^	^c^ RR
Chlorpyrifos	2018	179.75 (131.97–236.47)	841.11 (561.89–1673.56)	1.91 ± 0.29	1.48	2.08
	2019	102.02 (53.50–150.47)	886.51 (516.95–2733.01)	1.36 ± 0.28	1.67	1.18
	2020	56.77 (7.58–107.13)	1207.35 (513.66–28677.79)	0.96 ± 0.30	5.94	0.65
Methomyl	2018	141.32 (97.87–189.88)	764.40 (496.42–1647.43)	1.74 ± 0.29	1.42	1.07
	2019	369.00 (251.16–635.40)	3699.14 (1581.64–25705.64)	1.28 ± 0.26	1.71	2.279
	2020	155.66 (106.59–212.75)	956.87 (590.38–2341.14)	1.62 ± 0.28	3.65	1.18
Alpha-cypermethrin	2018	85.57 (62.31–114.94)	453.29 (287.13–1019.37)	1.77 ± 0.28	4.04	1.75
	2019	80.56 (59.92–105.68)	366.74 (245.51–723.72)	1.94 ± 0.29	0.64	1.65
	2020	24.37 (13.72–35.46)	197.58 (111.07–701.52)	1.41 ± 0.30	3.96	0.50
Hexaflumeron	2018	1.39 (0.41–2.99)	110.87 (35.99–1183.87)	0.67 ± 0.13	0.05	0.10
	2019	2.44 (0.62–5.49)	242.83 (74.26–3266.44)	0.64 ± 0.13	0.20	0.17
	2020	3.08 (0.57–7.88)	861.27 (166.27–70842.59)	0.52 ± 0.13	0.66	0.21
*B. thuringiensis*	2018	6.62 (1.38–16.50)	1643.06 (326.38–112702.92)	0.53 ± 0.13	0.05	1.08
	2019	8.59 (2.17–20.90)	1921.61 (378.38–122921.23)	0.54 ± 0.13	0.35	1.41
	2020	13.37 (4.77- 30.32)	1782.62 (403.77–55643.17)	0.60 ± 0.13	0.31	2.19
Spinosad	2018	0.030 (0.01–0.06)	2.12 (0.68–21.92)	0.69 ± 0.13	1.87	3.37
	2019	0.031 (0.01–0.07)	4.26 (1.05–108.98)	0.60 ± 0.13	0.32	3.48
	2020	0.025 (0.01–0.05)	1.51 (0.52–12.23)	0.72 ± 0.13	0.68	2.81

^a^: LC_50_: concentration causing 50% mortality. ^b^: LC_90_: concentration causing 90% mortality. ^c^: RR: LC_50_ value of the field strain/LC_50_ value of the susceptible strain.

**Table 6:** Carboxylesterases and AChE enzyme activity (mean ± SE) in field strains of *Spodoptera littoralis* over three consecutive seasons (2018 to 2020).

Strain	Mean ± SE								
	Carboxylesterases						AChE (µg AchBr/min/mg of Protein)		
	α-Esterases (µg α-Naphthol/min/mg of Protein)			β-Esterases (µg β-Naphthol/min/mg of Protein)					
	2018	2019	2020	2018	2019	2020	2018	2019	2020
Susceptible	25.36 ± 0.55			18.06 ± 0.39			9.63 ± 0.21		
Fayoum	24.56 ± 0.32	23.41 ± 0.73	20.25 ± 0.59 ***	17.49 ± 0.23	16.67 ± 0.52	14.42 ± 0.42 ***	9.33 ± 0.12	8.89 ± 0.27	7.69 ± 0.22 ***
Beheira	22.80 ± 0.18 **	21.80 ± 0.11 **	22.49 ± 0.18 *	16.23 ± 0.13 **	15.52 ± 0.08 **	16.01 ± 0.13 *	8.66 ± 0.06 **	8.28 ± 0.04 **	8.54 ± 0.07 *
Kafr El-Sheikh	20.65 ± 0.39 ***	23.48 ± 0.22	23.73 ± 0.61	14.70 ± 0.28 ***	16.72 ± 0.16	16.89 ± 0.43	7.84 ± 0.15 ***	8.92 ± 0.08	9.01 ± 0.23
F	28.85	9.28	17.32	28.85	9.28	17.32	28.85	9.28	17.32
*p*-value	0.0001	0.0055	0.0007	0.0001	0.0055	0.0007	0.0001	0.0055	0.0007

* Significantly different at *p* = 0.05. ** Significantly different at *p* = 0.01. *** Significantly different at *p* < 0.001

**Table 7:** MFO and GST enzyme activity (Mean±SE) in field strains of *Spodoptera littoralis* over three consecutive seasons (2018 to 2020).

Strain	Mean ± SE					
	MFO (mg/mg of Protein)			GST (mmol/min/mg of Protein)		
	2018	2019	2020	2018	2019	2020
Susceptible	1.47 ± 0.03			2.23 ± 0.04		
Fayoum	1.42 ± 0.01	1.35 ± 0.04	1.17 ± 0.03 ***	2.16 ± 0.02	2.06 ± 0.06	1.78 ± 0.05 ***
Beheira	1.32 ± 0.01 **	1.26 ± 0.006 **	1.30 ± 0.01 *	2.01 ± 0.01 **	1.92 ± 0.01 **	1.98 ± 0.01 *
Kafr El-Sheikh	1.19 ± 0.02 ***	1.36 ± 0.01	1.37 ± 0.03	1.82 ± 0.03 ***	2.07 ± 0.02	2.09 ± 0.05
F	28.85	9.28	17.32	28.85	9.28	17.32
*p*-value	0.0001	0.0055	0.0007	0.0001	0.0055	0.0007

* Significantly different at *p* = 0.05. ** Significantly different at *p* = 0.01.*** Significantly different at *p* < 0.001.

## Data Availability

All data of the study have been presented in the manuscript.

## Supplementary Materials

The following supporting information can be downloaded at: https://www.mdpi.com/article/10.3390/toxics11030211/s1, Table S1: Serial concentrations used in bioassay experiments.
