# Supplementary material for: Monitoring Resistance and Biochemical Studies of Three Egyptian Field Strains of Spodoptera littoralis (Lepidoptera: Noctuidae) to Six Insecticides
Source: Toxics. 2023 Feb 24;11(3):211. doi: 10.3390/toxics11030211 (PMC10053388; doi:10.3390/toxics11030211)
Supplement: Supplementary file 1 [file toxics-11-00211-s001.zip › toxics-2220416-supplementary.pdf]

Table S1. Serial concentrations used in bioassay experiments.

**Insecticides common name**	**Concentrations**
Chlorpyrifos	0.05, 0.025, 0.0125, 0.00625, 0.00312 and 0.00156 mg/L
Methomyl	2, 1, 0.5, 0.25, 0.125 and 0.0625 mg/L
Alpha-cypermethrin	4, 2,1, 0.5, 0.25 and 0.125 mg/L
Hexaflumuron	1, 0.25, 0.0625, 0.0156, 0.0039 and 0.00097 mg/L
Bacillus thuringiensis	2, 0.5, 0.125, 0.0312, 0.0078 and 0.0019 mg/L
Spinosad	0.025, 0.00625, 0.00156, 0.00039, 0.000098 and 0.000024 mg/L
